# Response Surface Optimization of Matched-Die Consolidation for BMI-Based CFRP Prepreg Laminates Toward Stiffened-Shell Manufacturing

**DOI:** 10.3390/polym18040483

**Published:** 2026-02-14

**Authors:** Bo Yu, Yinghao Dan, Haiyang Sun, Yu Kang, Bowen Zhang, Yuning Chen, Ziqiao Wang, Jiuqing Liu

**Affiliations:** 1 Northeast Forestry University Mechanical and Electrical Engineering College, Harbin 150040, China; yyyubbbo@nefu.edu.cn; 2 Harbin Fiberglass Research Institute Co., Ltd., Harbin 150029, China; sunhaiyang@harbinfrp.com (H.S.);; 3 National Key Laboratory of Test and Evaluation of ElectroMagnetic Sp, Luoyang 471000, China; dantesh@sjtu.edu.cn

**Keywords:** bismaleimide (BMI) resin, carbon fiber-reinforced polymer (CFRP), response surface methodology (RSM), tensile strength optimization, stiffened-shell structure

## Abstract

Hypersonic vehicles impose stringent requirements on lightweight structures to maintain mechanical integrity under extreme thermal environments. Bismaleimide (BMI)-based carbon fiber-reinforced polymer (CFRP) composites, featuring a high glass transition temperature and excellent thermal stability, are regarded as promising candidates for such applications. However, the high curing temperature and narrow processing window of BMI resins make it challenging to manufacture stiffened-shell structures with low defect levels and high fiber volume fractions. In this study, an integrated manufacturing route—hot-melt prepregging–filament winding–matched-metal mold forming—is proposed, and the key processing parameters are optimized via single-factor experiments and the Box–Behnken response surface methodology. The tensile strength of the laminate is selected as the response variable to evaluate the effects of the compression displacement (A), thermal consolidation/bonding temperature (B), heating rate (C), and cooling rate (D). The results reveal a unimodal dependence of the tensile strength on each parameter, with the significance ranking B > D > A > C; moreover, the A–B and A–D interactions are significant (*p* < 0.01). The established quadratic regression model exhibits good agreement with experimental data (R^2^ = 0.974; R^2^_adj = 0.949). The predicted optimum conditions are A = 0.07 mm, B = 114.93 °C, C = 1.35 °C·min^−1^, and D = 4.58 °C·min^−1^, corresponding to a predicted tensile strength of approximately 2287 MPa. Validation experiments yielded 2291 MPa, in excellent agreement with the prediction. Microstructural observations indicate tight interlaminar bonding and a pronounced reduction in voids under the optimized conditions. Applying the optimized process to fabricate stiffened-shell demonstrators achieves a fiber volume fraction of >60% and a void content of <1%. This work provides a quantitatively defined processing window and parameter optimization basis for the high-quality manufacturing of BMI-CFRP stiffened-shell structures, with significant engineering relevance.

## 1. Introduction

With the rapid advancement of aerospace technologies, hypersonic vehicles (Mach number > 5) have become a strategic research frontier worldwide [1]. During long-duration high-speed cruising in near space, these vehicles operate under an extremely severe “thermal barrier” environment. Intense aerodynamic heating can raise the stagnation temperature on the vehicle surface to above 1000 °C, while the service temperatures of large-area skins and primary load-bearing structures are often sustained in the range of 200–300 °C. Under such pronounced thermomechanical coupling, conventional aerospace aluminum alloys (e.g., 2024 and 7075 series) suffer sharp strength degradation due to high-temperature softening and can no longer satisfy the requirements of primary structures. Titanium alloys exhibit better heat resistance; however, their high density significantly constrains lightweight structural design, thereby penalizing the payload capacity and range. As hypersonic vehicles evolve toward larger scales, higher precision, lower weights, and higher reliability, composite stiffened shells, as major load-bearing components, are subject to increasingly stringent requirements for dimensional stability and mechanical performance in complex service environments [2].

Among the candidate materials, advanced fiber-reinforced polymer (FRP) composites—owing to their high specific strength, high specific modulus, and outstanding designability—have become a key development direction for hypersonic structural materials [3]. In particular, carbon fiber-reinforced polymers (CFRPs) offer high specific strength, high specific stiffness, and substantial design flexibility and are therefore widely used in critical structures such as fuselage frames, payload fairings, and cylindrical shells [4,5]. These materials provide irreplaceable advantages in weight reduction, fuel efficiency improvement, and maneuverability enhancement. Nevertheless, under high-temperature and hygrothermal service conditions, moisture uptake by the matrix may trigger residual-stress relaxation and volumetric swelling, leading to dimensional changes and property degradation; this remains one of the key issues limiting engineering applications [6].

The overall performance of composites at elevated temperatures is governed primarily by the heat resistance of the matrix resin [7]. Conventional epoxy systems typically exhibit a glass transition temperature (Tg) below 180 °C; at higher temperatures, they undergo pronounced mechanical property decay and thermo-oxidative degradation, making them unsuitable for long-duration hypersonic cruising conditions. To overcome this temperature limitation, bismaleimide (BMI) resins have attracted considerable attention as a new generation of high-temperature thermoset matrices [8]. BMI resins feature a favorable balance of mechanical properties, thermal stability, hygrothermal resistance, radiation resistance, and good processability. Accordingly, BMI resins are regarded as ideal candidate matrices for advanced high-temperature aerospace composite structures [9] and have been applied in both primary and secondary load-bearing components, including wing skins, horizontal and vertical tails, and fuselage structures [10].

However, BMI systems also exhibit inherent processing constraints. BMI monomers have high melting points and yield high crosslink densities after curing, resulting in relatively high brittleness and limited impact damage tolerance. Moreover, BMI viscosity is highly temperature-sensitive, leading to a narrow processing window, while the curing temperature is generally high (typically above 170–200 °C). During cooling, mismatch in the coefficients of thermal expansion (CTEs) between carbon fibers and the resin matrix can generate substantial thermal residual stresses, which may induce matrix microcracking and consequently degrade the interlaminar shear strength and compressive performance. Therefore, achieving both a high-temperature capability and robust forming quality with dimensional stability for BMI composite structures remains a key challenge in engineering practice [11].

For complex thin-walled load-bearing structures, such as composite stiffened shells commonly used in hypersonic vehicles, conventional manufacturing relies primarily on autoclave processing [12]. Although autoclaves can provide relatively uniform temperature and pressure fields, they are associated with high equipment costs, high energy consumption, and long processing cycles. To address these limitations, integrating prepreg-based processing with filament winding and matched-metal-die forming offers a viable route for producing stiffened shells with high fiber volume fractions, high dimensional accuracy, and low defect contents [13,14]. Specifically, prepreg processing ensures uniform resin distribution and adequate fiber impregnation; filament winding enables the accurate and efficient layup of complex grid-stiffener architectures [15]; the matched-metal-die-forming process utilizes the closure of a rigid core and cavity to apply mechanical pressure to the composite material preform [16]. It has significant advantages, such as double-sided precision control, high-pressure densification, and efficient heat transfer. Therefore, introducing the matched-metal-die process into the preparation of BMI/CFRP reinforced shell is of great engineering significance for achieving high dimensional accuracy and high forming quality. It should be noted that matched-metal-die forming involves multiple process parameters—such as the compression displacement, thermal bonding (hot-press) temperature, heating rate, and cooling rate—which exhibit complex coupled effects [17,18]. Improper control can readily cause manufacturing defects, including resin-starved regions, resin-rich zones, fiber buckling, stress concentration, or shell warpage, thereby reducing the load-bearing capability and service reliability. Beyond the integration of the process route, the systematic optimization of the key processing parameters is essential for obtaining high-performance composite stiffened shells.

The response surface methodology (RSM) enables the quantitative evaluation of the main effects and interactions among multiple factors with a limited number of experiments, providing an effective tool for identifying complex processing windows and parameter prediction [19,20]. On this basis, this work proposes and optimizes a “hot-melt-prepreg-processing–filament winding–matched-metal-die-forming” process for BMI–CFRP stiffened shells and establishes a closed-loop research framework of “process window calibration–statistical modeling–mechanistic substantiation–engineering validation.” Specifically, hot-melt prepreg processing is employed to achieve the uniform impregnation of carbon fiber tows by BMI resin; filament winding is used to fabricate a high-fiber-volume-fraction stiffener preform on a cylindrical mandrel; subsequently, unidirectional laminates are adopted as carriers for process window definition and defect sensitivity assessment, enabling preliminary experiments and single-factor studies to screen the key parameter ranges. A Box–Behnken experimental design is then applied to develop an RSM model using the laminate tensile strength as the response, systematically revealing the main and interactive effects of the compression displacement, thermal bonding temperature, heating rate, and cooling rate, yielding a globally optimal parameter set. On this basis, microscopic morphology characterization and thermomechanical analyses are conducted to elucidate the mechanisms associated with the defect suppression and residual-stress evolution. Finally, the optimized parameters are transferred to the fabrication of representative stiffened-shell specimens, and the fiber volume fraction, porosity level, dimensional accuracy, and property retention under high-temperature/hygrothermal conditions are comprehensively evaluated to validate the engineering applicability of the proposed process strategy.

## 2. Materials and Methods

### 2.1. Materials

The reinforcement used in this study was T880-grade carbon fiber (SYT55G), supplied by Harbin FRP Research Institute Co., Ltd. Harbin, China. The T880-grade carbon fibers were used with the standard commercial sizing agent provided by the supplier. Before usage, the fibers were subjected to a drying process to remove adsorbed moisture, as detailed in Section 2.3. This fiber is classified as an intermediate-modulus, high-strength carbon fiber (Intermediate Modulus, High Strength). Its typical properties include a tensile strength of ≥5.5 GPa, a tensile modulus of approximately 290 GPa, an elongation at break of ~1.9%, and a linear density of ~445 g/km (6K tow). Compared with widely used high-strength carbon fibers such as T300 and T700, T880 exhibits a higher elastic modulus and tensile strength, thereby markedly improving the specific stiffness and specific strength of the resulting composites while maintaining low density. This is beneficial for suppressing the buckling instability of thin-walled composite stiffened shells under high aerodynamic loads.

**Figure 1:**
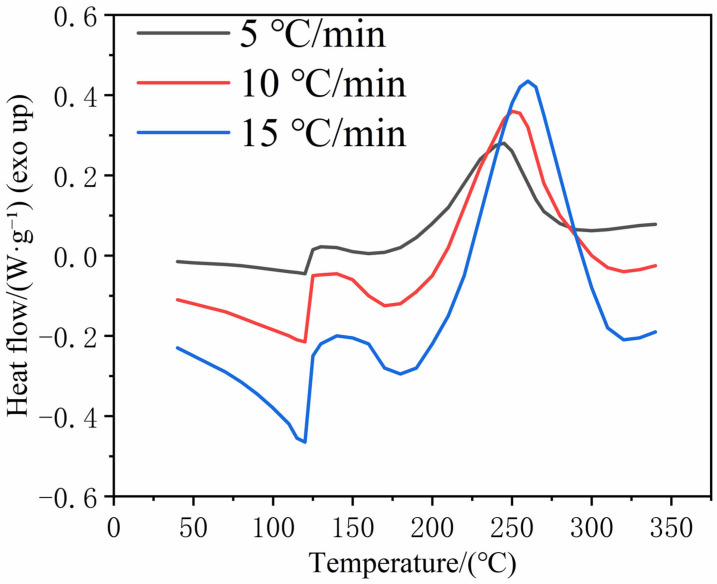
DSC curves of bismaleimide resin at different heating rates.

The matrix was a bismaleimide resin system (Bismaleimide, BMI), QY8911, also provided by Harbin FRP Research Institute Co., Ltd. This resin system is a two-component modified BMI system primarily composed of 4,4′-bismaleimidodiphenylmethane (BDM) and 2,2′-diallylbisphenol A (DABPA). Specifically, BDM serves as the main thermosetting resin monomer providing high heat resistance, while DABPA acts as a reactive toughening modifier (copolymer) to improve the processability and toughness of the cured network. The polymerization involves the Diels–Alder reaction and Ene reaction between the maleimide groups of BDM and the allyl groups of DABPA. BMI resins are high-performance thermosetting polymers; their molecular backbone contains benzene rings and rigid imide ring structures, which enable a high glass transition temperature (Tg) of up to 280 °C. In addition, BMI resins offer favorable hygrothermal resistance, dimensional stability, flame retardancy, and dielectric stability. Differential scanning calorimetry (DSC) was conducted using a DSC8000 instrument (PerkinElmer, Rodgau, Germany), and DSC curves were obtained at different heating rates, as shown in Figure 1. With an increasing heating rate, the characteristic peaks of the curing reaction shift toward higher temperatures. Taking the curve at 10 °C/min as an example, an endothermic peak appears at ~130 °C, corresponding to the dissolution of BDM in DABPA. A smaller exothermic peak is observed at ~140 °C, attributable to the Diels–Alder addition reaction between BDM and DABPA. The main exothermic peak near ~260 °C corresponds to the exothermic heat release associated with the curing and crosslinking reaction of the resin, and its peak temperature represents the characteristic curing exotherm of the BMI resin system.

Overall, the combination of T880-grade carbon fiber and BMI resin outperforms conventional “epoxy + T300/T700” systems in terms of its high-temperature load-bearing capability, specific strength/specific stiffness, and hygrothermal stability. Compared with “polyimide + high-modulus carbon fiber” systems, it also offers more favorable processability and cost advantages. Therefore, the T880/BMI system was selected as a representative material for composite stiffened shells in hypersonic vehicles, balancing performance requirements with engineering manufacturability and practical feasibility.

### 2.2. The Theoretical Basis of the Forming Process

Before conducting the “hot-melt pre-impregnation—fiber-winding—metal-mold-forming” process and parameter optimization, it is necessary to systematically analyze the key mechanisms of the BMI resin hot-bonding/molding process from three aspects: the chemical rheology of the curing, the compaction and permeation of the preform, and the evolution of the thermal residual stress. The core lies in the fact that the viscosity of BMI resin changes in a coupled manner with the temperature and curing degree, determining the “flowable—exhaustable—penetrable” time window; the change in the compression amount alters the pressure gradient and permeability, affecting the permeation of the resin in the fiber bed and the elimination of pores; the cooling rate determines whether the stress relaxation near Tg is sufficient, thereby influencing the residual stress and interlayer damage sensitivity. Based on these mechanisms, this study selects the compression amount (A), hot-bonding temperature (B), heating rate (C), and cooling rate (D) as the four key factors for response surface optimization and emphasizes their strong coupling characteristics.

#### 2.2.1. Chemorheological Behavior of BMI Resin and Viscosity Window Control

The chemical rheology and flow behavior of viscosity-controlling resins are the key factors determining the molding quality of composite materials. The viscosity (η) of BMI resin is a function of the temperature (T) and curing degree (α) and follows the double Arrhenius equation model:

(1) $$\ln\eta \left(T,\alpha \right)=\ln\eta_{0}+\frac{E_{\eta }}{RT}+K_{\eta }\alpha \exp\left(\frac{E_{k}}{RT}\right)$$

where η_0_ is the pre-factor, E_η_ is the viscosity activation energy of the viscous flow, E_k_ is the activation energy of the curing reaction, and R is the gas constant. In the initial stage of temperature increase (the low-α phase), the increase in temperature enhances the movement of molecular chains, and the viscosity (η) decreases rapidly with the increase in temperature, which is conducive to the infiltration of the resin between carbon fiber bundles and the expulsion of interlayer bubbles; during the stage where the heating temperature is maintained, the curing reaction accelerates, the crosslinking density increases rapidly, and the increase in α leads to an exponential increase in viscosity, approaching the gel point. The time interval from the significant decrease in viscosity to before the gel point constitutes the optimal process window for resin flow, bubble elimination, and infiltration. The heating temperature (B) and heating rate (C) determine the width and position of this “minimum viscosity—curing” window: when B is too low, the viscosity of the system is too high, infiltration is insufficient, and it is prone to producing dry spots and air entrapment; when B is too high or C is too large, the system quickly crosses the low-viscosity zone and enters the gel zone, and crosslinking occurs before the flow and bubble elimination are completed, which is prone to causing short-shot and poor interlayer-bonding defects.

#### 2.2.2. Theory of Preform Compression Displacement

During hot-melt prepregging and die-closing compaction, the permeation of BMI resin within the carbon fiber preform can be approximated as viscous flow in a porous medium. The average velocity (*v*) can be described by Darcy’s law:

(2) $$\nu =-\frac{K}{\mu }\nabla P$$

where v is the resin flow velocity, K is the permeability of the fiber preform, μ is the resin viscosity, and ∇P is the pressure gradient. Increasing the compression displacement (factor A) essentially increases the effective compaction pressure in the mold cavity (i.e., ∇P), thereby enhancing the driving force for resin flow, which is beneficial for bubble removal and increasing the fiber volume fraction. Meanwhile, however, the permeability (K) of the fiber bed decreases significantly with increasing compression displacement. According to the qualitative relationship given by the Kozeny–Carman equation:

(3) $$K\propto \frac{\varepsilon^{3}}{{\left(1-\varepsilon \right)}^{2}}$$

where ε is the inter-fiber porosity. When the compression displacement (A) is excessive, the reduction in ε causes a sharp decrease in K; the resin flow channels are compressed or even locally closed, which may instead lead to regions where the resin supply becomes insufficient, inducing defects such as resin-starved areas, dry spots, or closed porosity. Therefore, the compression displacement (A) must be coordinated with the resin viscosity window (controlled by B and C): effective compaction and degassing should be completed as much as possible during the low-viscosity stage, and excessive compaction near gelation—when the resin viscosity is high—should be avoided to prevent “resin squeeze-out” and fiber buckling.

#### 2.2.3. Thermal Residual Stress and Cooling-Rate Theory

The cooling rate (factor D) is another key factor affecting the mechanical performance of high-temperature composites. BMI/CF composites consist of anisotropic carbon fibers with an extremely small longitudinal coefficient of thermal expansion and an isotropic BMI resin matrix with a relatively large coefficient of thermal expansion. The longitudinal coefficient of thermal expansion (CTE) of carbon fiber is: $\alpha_{f}\approx -0.5\times 10^{-6}/{}^{\circ}C$. The CTE of BMI resin is: $\alpha_{m}\approx 40–60\times 10^{-6}/{}^{\circ}C$. During cooling from the curing temperature (T_c_) to room temperature (T_r_), the axial dimension of the composite is essentially “locked” by the carbon fibers; consequently, the free shrinkage of the resin phase is constrained, generating tensile residual stress in the matrix (δ_res_). This can be expressed in a simplified form as Equation (4):

(4) $$\delta_{\mathrm{res}}\propto \int_{T_{room}}^{T_{cure}}\left(\alpha_{m}-\alpha_{f}\right)E_{m}\left(T\right)dT$$

where δ_res_ is the equivalent residual stress in the matrix, E_m_ is the equivalent modulus of the matrix, and α_m_ and α_f_ are the linear coefficients of the thermal expansion of the BMI matrix and carbon fiber, respectively.

The cooling rate (D) has a decisive influence on the level and distribution of residual stress. If the cooling is too fast, the system spends insufficient time near the Tg, making it difficult for the resin chain segments to relax, and the thermal strain is “frozen” in the cured material, resulting in an increase in residual stress. At the microscopic level, this often manifests as matrix bending cracks, interface debonding, and interlayer microcracks, which eventually lead to stress concentration under tensile/compressive loads, weakening the interlayer shear strength and compressive performance. On the contrary, using an appropriate cooling rate and maintaining a certain time at temperatures above and near the Tg can utilize the viscoelastic relaxation of the resin to release part of the thermal strain, thereby reducing the final residual stress. Therefore, D needs to be balanced between the production cycle and stress control, and its reasonable range should be determined by combining single-factor and response surface experiments.

In summary, the compression amount (A), heating sealing temperature (B), heating rate (C), and cooling rate (D) jointly dominate the forming quality of the molded-in-plane single-layer plate produced by compression molding. Considering the strong temperature/curing-degree dependence of viscosity (μ(T,α)), there is significant coupling among the four factors. This study narrowed the parameter range through single-factor pre-experiments and used the response surface method to analyze the interaction between A and D to determine the optimal process combination: at the stage with the lowest viscosity and best fluidity, an appropriate compression amount should be used to fully exhaust the air and increase the fiber volume fraction; excessive pressing in the high-viscosity stage should be avoided to reduce the risks of fiber buckling, the resin “drying out”, and pore sealing.

### 2.3. Forming Process of BMI–CFRP Unidirectional Laminates

**Figure 2:**
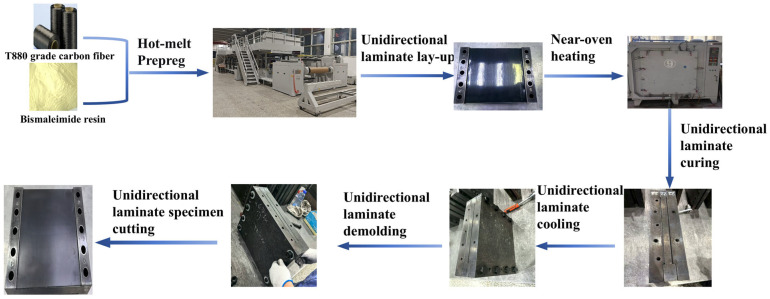
The process flow of the one-way plate-forming method using BMI-CFRP.

In this study, the abovementioned BMI resin system and T880-grade carbon fiber were used to prepare BMI/CF prepreg and BMI–CFRP unidirectional laminates, which served as specimens for the process window calibration, microstructural characterization, and mechanical testing, as well as for the subsequent fabrication of stiffened shells. The overall preparation route is shown in Figure 2, and the detailed procedures are described as follows.

(1) Preparation of BMI/CF Prepreg

Carbon fiber (CF) was placed in a ventilated environment at 105 °C for 6 h to remove moisture adsorbed on the fiber surface. The BMI resin, kept in a sealed condition, was removed and left at room temperature for 24 h. The resin was then unsealed and heated at 80 °C until it was fully molten. A resin film was prepared using a film-coating machine; the coating temperature, coating speed, and coating pressure were adjusted to ensure uniform film formation on the release paper. The resin film was collected using a winder and stored.

The BMI/CF prepreg was fabricated using a commercial hot-melt prepreg production line (Model 1270R, Juhe Xing Enterprise Co., Ltd., Shanghai, China). As shown in Figure 2, this industrial-scale equipment consists of a high-precision resin film-coating unit and multi-stage impregnation unit.

The key technical specifications include:

The Coating System: The coating system has an effective width of 1270 mm with a roller gap adjustment precision of 0.001 mm and temperature control accuracy of ±1 °C, ensuring the preparation of resin films with uniform thickness and areal weight.

The Impregnation System: The impregnation system has a creel capacity of 600 spools with a constant-tension control system to maintain fiber alignment. The compaction unit features five sets of hot-pressing rollers, allowing for gradual and thorough resin infiltration into the fiber tows under controlled pressure and temperature (up to 120 °C).

The impregnation temperature, speed, and pressure of the prepreg machine were adjusted (Table 1). CF was then combined with the BMI resin film, allowing the BMI resin to uniformly encapsulate the fibers while maintaining fiber straightness and uniform distribution, thereby obtaining the BMI/CF prepreg. The prepreg thickness and surface quality were inspected. The BMI/CF prepreg was sealed in a plastic vacuum bag and stored at −18 °C until use.

(2) Fabrication of BMI–CFRP Unidirectional Laminates

The sealed BMI/CF prepreg was removed from the −18 °C freezer and kept at room temperature for 24 h to fully equilibrate. It was then unsealed and cut into 600 mm × 600 mm sheets using a cutting machine. The unidirectional laminate was formed using a matched-metal-die set (female die/male die). A mold release agent was applied to the die surfaces twice, with an interval of no less than 30 min between applications.

The prepreg plies were laid up layer by layer with the same fiber orientation. Each layer was debulked/compacted and degassed to remove entrapped interlaminar air and ensure layup densification. After 14 plies were stacked, the mold was assembled and placed on a hot press for die closing. Under the press load, displacement control was used to achieve the prescribed compression displacement (A). During the hot-bonding stage, a constant compression displacement was maintained to regulate the final fiber volume fraction and resin distribution.

The curing process consisted of four stages: “heating–hot bonding–post-curing–cooling”. First, the temperature was increased from room temperature to the thermal bonding temperature (B) at the prescribed heating rate (C) to promote impregnation and degassing within the resin flow window where viscosity decreases significantly. The laminate was then held at B for 2 h while keeping the compression displacement (A) unchanged, enabling sufficient fiber wet-out, void removal, and initial crosslinking. Subsequently, the temperature was further increased to 170 °C and held for 4 h for post-curing to achieve deeper crosslinking and enhance the thermal deformation resistance and hygrothermal stability. Finally, the laminate was cooled at the prescribed cooling rate (D) to below 60 °C, followed by unloading and demolding. After curing, the laminate was removed, trimmed to eliminate excess edge material, labeled, and reserved as BMI–CFRP unidirectional laminate specimens.

(3) Preparation of Specimens for Mechanical Testing

After curing and cooling to a safe demolding temperature, the mold was disassembled, and the unidirectional laminate was removed. Edge flash was trimmed, and the laminate thickness and surface appearance were measured. The internal quality of the laminate was evaluated by C-scan inspection to confirm the absence of defects, such as delamination. The qualified laminates were then machined into standard tensile specimens using a CNC machine (JDPDES_400, Beijing Jingdiao Group Co., Ltd., Beijing, China). Specimens with dimensions close to the Type I geometry specified in GB/T 1040.1–2008 were adopted, with a nominal size of 150 mm × 10 mm × 4 mm.

### 2.4. Preliminary Experiments and Single-Factor Experiments

The process parameters, including the compression displacement (A), thermal bonding temperature (B), heating rate (C), and cooling rate (D), have significant effects on the microstructural forming quality and tensile strength of BMI-based unidirectional laminates. To define reasonable parameter ranges prior to response surface optimization, preliminary experiments and single-factor experiments were first conducted to screen the key processing window.

(1) Gel time test

A standard gel time test (Gel Time Test) was performed to measure the stringing time of the BMI resin at 80 °C, 100 °C, and 120 °C, thereby characterizing the relationship between the available flow time and curing-rate evolution under different temperature conditions. The results showed that the gel time at 120 °C was approximately 40–50 min, which provides a relatively sufficient low-viscosity period for resin flow and impregnation while maintaining a moderate reaction advancement rate; therefore, 120 °C was selected as the high-level value of the thermal bonding temperature. At 80 °C, the resin viscosity was higher, and the gel time was significantly prolonged, indicating insufficient flowability; nevertheless, it was retained as the low-level temperature. The intermediate level was set at 100 °C for the subsequent graded parameter settings.

(2) Thickness compression pre-test

With the heating rate (C) and cooling rate (D) kept constant, unidirectional laminates were fabricated by varying the compression displacement (A) from 0.05 to 0.25 mm. Changes in the laminate thickness, resin overflow behavior, and fiber morphology were examined to identify the boundary conditions of the typical forming defects associated with excessive compaction. The results indicated that when A increased to 0.25 mm, substantial resin overflow occurred, “dry spots” appeared on the laminate surface, and wavy fiber distortion was observed; meanwhile, the porosity increased, suggesting that excessive compaction disrupts rational resin distribution and induces structural defects. Based on these observations, the upper limit of A was restricted to 0.15 mm, and the range of 0.05–0.15 mm was selected as the focus interval for the subsequent single-factor and response surface experiments.

(3) Design of single-factor experiments

To further clarify the effects of the key parameters in the hot-melt prepreg process on the tensile strength and forming quality of the unidirectional laminates, and to provide stable and reasonable parameter boundaries and trend information for response surface optimization, four sets of single-factor experiments were designed following the principle of “vary one factor while fixing the other factors at their central levels,” based on the ranges determined in the preliminary experiments. The parameter combinations for these four groups—the compression displacement (A), thermal bonding temperature (B), heating rate (C), and cooling rate (D)—are listed in Table 2. For each parameter combination, at least three unidirectional laminates were fabricated and machined into tensile specimens for testing in order to reduce scatter and ensure the statistical reliability of the results.

### 2.5. Response Surface Experiments

Based on the single-factor experiments, in order to systematically investigate the interaction effects of the four process parameters: the compression amount (A), heat-sealing temperature (B), heating rate (C), and cooling rate (D), and to obtain the optimal combination for the tensile strength of the unidirectional plate, this study employed Box–Behnken design (BBD) response surface optimization. Taking A, B, C, and D as the independent variables and the 0° tensile strength (σt) of the BMI-CFRP unidirectional plate as the response value (Y), the response surface optimization was conducted. A quadratic regression model with four factors and three levels was constructed. Table 3 lists the test factor levels, with a total of 29 test points. Compared with the central composite design (CCD), the use of Box–Behnken design (BBD) avoids the combination of extreme points (such as the highest-temperature + highest-pressure group), and the experimental safety is higher.

The form of the quadratic polynomial regression model is Equation (5):

(5) $$Y=\beta_{0}+\sum_{i=1}^{4}\beta_{i}X_{i}+\sum_{i=1}^{4}\beta_{ii}X_{i}^{2}+\sum_{1\leq i<j\leq 4}\beta_{ij}X_{i}X_{j}$$

In Design-Expert, the data was fitted, and variance analysis was conducted to obtain the significance (F values, *p* values) of the main effects, quadratic terms and interaction terms of each factor. Based on this, response surface and contour plots were drawn to analyze the coupling laws of process and performance, and the optimal process parameters and their predicted tensile strength were obtained through the optimization module. Finally, a verification test was conducted under the predicted optimal parameters, and the measured values were compared with the predicted values to evaluate the reliability of the model.

### 2.6. Mechanical Performance Characterization

Tensile tests were conducted using an electronic universal testing machine (INSTRON 5966, Instron Corporation, Norwood, MA, UK). The test environment was at room temperature (approximately 25 °C), and a constant-displacement control mode was adopted. The crosshead speed was 10 mm/min. The test used a sample of a similar size to Type I in GB/T 1040.1-2008 [21], with a standard sample size of 150 mm × 10 mm × 4 mm. The sample was cut along the single-directional board fiber direction, ensuring that the tensile direction was parallel to the fiber direction. Each experiment was repeated at least five times to ensure stability and accuracy.

The tensile strength was calculated using Equation (6):

(6) $$\sigma_{t}=\frac{F_{\max}}{b\cdot h}$$

The bending performance test is conducted using the three-point bending method, in accordance with the standard GB/T 9341-2008 [22]: Determination of bending performance of plastics. The standard sample size is 80.0 mm × 10.0 mm × 4.0 mm. The bending strength can be calculated using Equation (7):

(7) $$\sigma_{f}=\frac{3F_{\max}L}{2bh^{2}}$$

where σ_t_ represents the tensile strength (MPa), σ_f_ represents the bending strength (MPa), F_max_ represents the maximum load (N), b represents the width of the specimen (mm), and h represents the thickness of the specimen (mm). At least 5 parallel specimens were tested for each process combination, and the average value was taken along with the standard deviation. The longitudinal tensile modulus (E_t_) was estimated based on the slope of the linear segment of the stress–strain curve.

Dynamic mechanical analysis was performed using a DMA Q850 (TA Instruments, New Castle, DE, USA). The dynamic storage modulus and loss modulus of rectangular composite laminates with dimensions of 30 mm × 5 mm × 2.2 mm were tested under a heating rate of 3 °C/min, a fixed frequency of 1 Hz, and an amplitude of 0.006 mm using the single-cantilever method.

## 3. Results

### 3.1. Microstructure Analysis and Process Discussion

**Figure 3:**
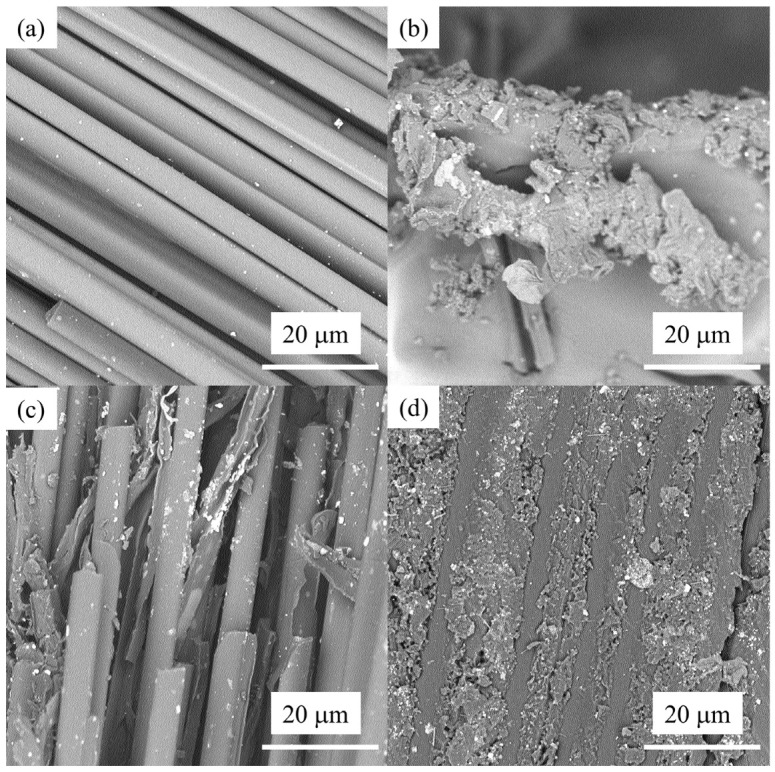
(**a**) An SEM image of the surface morphology of a single carbon fiber bundle; (**b**) an SEM image of BMI dual resin; (**c**) an SEM image of the BMI/CF pre-impregnated material after the hot-melt pre-impregnation process; (**d**) an SEM image of the BMI-CFRP unidirectional plate after metal mold compression curing.

Microstructural features were examined using scanning electron microscopy (SEM, Phenom ProX, Phenom-World B.V., Eindhoven, The Netherlands). In Figure 3, representative SEM images are provided for the carbon fibers, cured BMI resin, BMI/CF prepreg produced by the hot-melt impregnation process, and BMI-CFRP unidirectional laminate.

Figure 3a shows the SEM image of T880-grade carbon fibers. The filaments are clearly distinguishable and relatively well aligned, with an average single-fiber diameter of approximately 5 μm. The fiber surface appears straight and smooth, and no obvious surface flaws are observed. The fracture surface exhibits typical brittle fracture characteristics, without noticeable plastic necking or fibrillar drawing, indicating good intrinsic fiber quality and that failure is dominated by brittle mechanisms. Figure 3b presents the surface morphology of the cured bismaleimide (BMI) resin. The resin phase shows continuous flow-like textures with locally agglomerated blocky features, suggesting that BMI possesses adequate flowability during the hot-melt and curing stages and can form a continuous matrix phase. No apparent through-thickness pores or large voids are observed, indicating that the cured resin is generally dense under the applied curing conditions, thereby providing a favorable matrix basis for subsequent fiber wetting and encapsulation.

Figure 3c shows the microstructure of the BMI/CF prepreg after the hot-melt impregnation process only. Compared with Figure 3a,b, an evident BMI resin layer is present on the fiber surfaces, and partial resin bridging between adjacent filaments can be observed. However, a considerable number of low-brightness regions and irregular cavities remain between the fiber bundles, with relatively large pore sizes. This indicates that although preliminary wetting and resin transfer occur during the impregnation stage, the overall consolidation remains insufficient, and the resin does not fully fill the inter-fiber spaces. Therefore, hot-melt impregnation alone is inadequate to achieve a dense composite structure. Figure 3d depicts the morphology of the BMI-CFRP unidirectional laminate fabricated via the matched-metal-die-molding process. The fiber bundles are clearly compacted and arranged in a highly parallel manner. A continuous BMI matrix tightly encapsulates the fibers and fills the inter-fiber gaps. The fiber volume fraction is approximately 62%, and the overall morphology is continuous and dense, with a porosity of about 0.8%. Almost no macroscopic voids or unimpregnated regions are observed. The fiber/matrix interfacial transition is smooth and well bonded, demonstrating that, under the optimized compression displacement and thermal schedule, matched-die molding effectively eliminates the large-void defects present in the prepreg state, markedly improving the laminate densification and interfacial quality and thereby providing a robust microstructural foundation for enhanced mechanical performance. The SEM observations further indicate that the processing parameters exert a direct influence on the microstructural quality of the composite.

### 3.2. Results of Single-Factor Experiment

**Figure 4:**
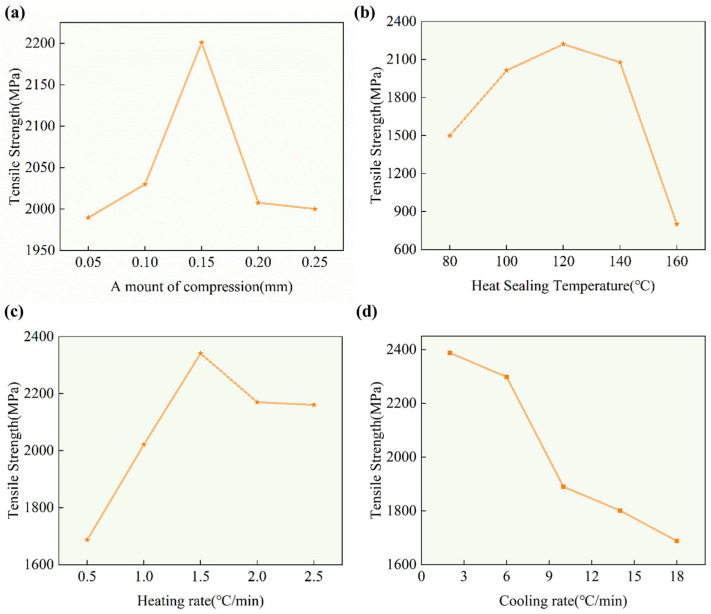
Effects of single-factor processing parameters on the tensile strength of BMI–CFRP unidirectional laminates: (**a**) compression displacement; (**b**) hot-press bonding temperature; (**c**) heating rate; (**d**) cooling rate.

A single-factor experiment was conducted to investigate the effects of the compression amount, heat-bonding temperature, heating rate and ply thickness on the tensile strength of the BMI-CFRP unidirectional plate. The results are shown in Figure 4a. The tensile strength of the BMI-CFRP unidirectional plate significantly increased with the increase in the compression amount, reaching a peak at 0.15 mm, with a tensile strength of 2200.91 MPa. Beyond this point, further increase in the compression amount led to a decrease in the tensile strength. This is because in the low-compression-amount stage, increasing the compression amount helps to force the viscous resin to flow between and around the carbon fibers, eliminating air and volatiles between the layers and reducing the porosity, allowing the fibers to be better impregnated by the resin.

Meanwhile, the compression amount causes the pre-impregnated layer to come into close contact with the layer. Under heating conditions, the resin diffuses and entangles across the interface, forming a strong interlaminar bond. An appropriate compression amount can optimize the fiber volume content of the composite material, allowing it to reach the optimal value, thereby maximizing the mechanical properties. At a compression amount of 0.15 mm, the tensile strength reaches its peak. At this point, the resin fluidity, air pore exclusion and interlaminar bonding reach an optimal balance state. The fiber volume content is also within the ideal range. When the compression amount continues to increase, excessive resin is squeezed out from the laminated plate, resulting in a “poor resin” area. This causes the fibers to be locally exposed, the bonding force to decrease, and a stress concentration point to form, causing brittle carbon fibers to collapse or bend, generating micro-damage, directly weakening the bearing capacity of the composite material and thereby reducing the tensile strength.

As shown in Figure 4b, the tensile strength of the BMI-CFRP unidirectional plate increases first and then decreases with the increase in the heat-bonding temperature. It reaches the peak at 120 °C, which is 2221.01 Mpa. This might be because during the low-temperature stage, the curing reaction and flow behavior of the double-matrix resin are highly sensitive to temperature. As the temperature rises, the resin viscosity significantly decreases, and the fluidity increases. This greatly promotes the diffusion, interweaving and healing of the resin between layers, forming a strong interface. The first stage of the curing temperature of the double-matrix resin curing system is 120 °C. At this temperature, the resin begins to undergo crosslinking reactions, achieving initial curing and obtaining strength. At 120 °C, the resin viscosity reaches the optimal process window, which not only ensures sufficient fluidity for healing the interface but also does not cause resin loss due to excessively low viscosity. During the high-temperature stage, the resin degrades or undergoes excessive pre-curing; thus, effective interlayer healing is prevented, leaving a weak interface. Moreover, an excessively high curing temperature increases the thermal stress during the curing process and leaves larger internal stress after cooling, thereby reducing the effective load-bearing capacity of the material. Therefore, the heat-bonding temperature is 120 °C, which matches the first-stage temperature of the curing system. As shown in Figure 4c, the tensile strength of the BMI-CFRP unidirectional plate increases first with the increase in the heating rate, reaches the peak at 1.5 °C/min, which is 2341.1 Mpa, and then decreases as the heating rate increases. This is because during the slow heating stage (0.5–1.5 °C/min), as the heating rate is appropriately increased, the temperature field in the mold and composite material becomes more uniform, avoiding an excessive temperature difference between the surface and core layer. The resin can simultaneously reach the low-viscosity stage, which is conducive to achieving uniform interlayer healing within the pressure window, and has sufficient time for the air and volatile components in the prepreg to slowly escape before the resin gels, reducing pores. However, excessively rapid heating will cause a significant temperature gradient in the thickness direction of the material. The outside is first heated and solidified, while the inside remains in a low-temperature state, resulting in asynchronous curing and generating large internal stress and microcracks. Moreover, volatile components rapidly vaporize and cannot be expelled in time, easily forming pores and bubbles, resulting in a decrease in tensile strength. As shown in Figure 4d, the tensile strength of the BMI-CFRP unidirectional plate significantly decreases with the increase in the cooling rate. This is because the increase in the cooling rate “locks” a large amount of thermal stress in the composite material, significantly increasing brittleness, and may trigger microcracks. The cooling rates of the core layer and surface layer are different, resulting in an uneven curing-degree distribution and affecting the overall performance, leading to a deterioration in the mechanical properties.

### 3.3. Response Surface Model Establishment and Significance Analysis

Table 4 presents the 29 sets of test results obtained based on the Box–Behnken test scheme listed in Table 3. Taking the compression amount (A), heating sealing temperature (B), heating rate (C), and cooling rate (D) as the independent variables, and the tensile strength (Y) of the BMI-CFRP single plate as the response value, the test data were subjected to quadratic polynomial regression fitting using Design-Expert 8.0.6.1 software. The empirical model between the tensile strength and each process parameter is as Equation (8):

(8) $$\begin{array}{l} Y=-18548.54+31478.13A+303.06B+2665.24C+350.54D-210.70AB\\ -2447.60AC+1083.21AD-7.33BC-1.79BD-38.37CD-60859.13A^{2}\\ -1.16B^{2}-742.74C^{2}25.88D^{2} \end{array}$$

where Y represents the tensile strength (MPa), while A, B, C, and D are the encoded process factors.

A variance analysis was conducted on the data in Table 4, and the results are shown in Table 5. The total F value of the model was 38.10, and the corresponding *p* value was 0.000 < 0.001, indicating that the quadratic polynomial model was extremely significant overall and could effectively describe the response relationship of the tensile strength to each process parameter. The *p* value of the misfit term was 0.5021 > 0.05, indicating that the misfit of the model was not significant and the residuals mainly originated from random errors rather than systematic errors, and the model had good statistical reliability. The model coefficient of determination (R^2^) was 0.974, and the adjusted coefficient of determination (R^2^Adj) was 0.949, indicating that approximately 94.9% of the variation in the tensile strength could be explained by the four process parameters and their interactions and quadratic terms. The fitting accuracy was high, and it could be used for process optimization and prediction.

From the significance of each item, the *p* values of the first-order terms, A, B, and D, were all less than 0.01, indicating that the main effects of the compression amount, heating sealing temperature, and cooling rate on the tensile strength were extremely significant; the *p* value of the C term (heating rate) was 0.7093, which was greater than 0.05, indicating that within the range of the investigated parameters, the first-order linear effect of the heating rate was relatively weak. In the interaction terms, the *p* values of AB and AD were 0.0052 and 0.0043, respectively, both less than 0.01, indicating that there was a strong interaction between the compression amount and heating sealing temperature, and between the compression amount and cooling rate; the *p* value of the BD term was 0.0411 < 0.05, also having a significant impact on the tensile strength, while the interaction terms AC, BC, and CD were not significant. The *p* values of the quadratic terms A^2^, B^2^, C^2^, and D^2^ were all less than 0.01, indicating that the tensile strength showed a clear non-linear “single-peak” characteristic with respect to each factor, and there was a clear optimal process window. By combining the magnitudes of the F values of each factor, the order of influence can be further determined: the F value of the heating sealing temperature (B) was the largest, followed by the cooling rate (D) and compression amount (A), and the F value of the heating rate (C) was the smallest. It can be seen that the heating sealing temperature was the most important process parameter determining the tensile strength, followed by the cooling rate and compression amount, while the influence of the heating rate within the range of this experiment was relatively secondary. This result was also consistent with the trend obtained from the single-factor experiments in the previous text, providing a basis for the subsequent response surface morphology analysis and process optimization.

**Figure 5:**
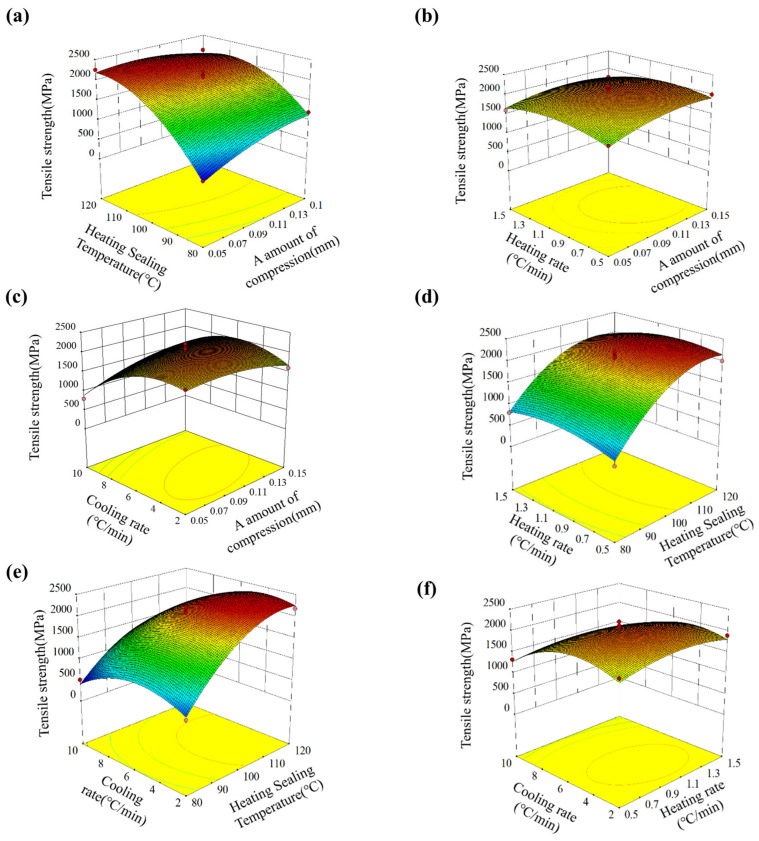
Response surface plots showing the interaction effects of processing parameters on the bending strength of BMI–CFRP laminates: (**a**) compression amount × heating sealing temperature; (**b**) compression amount × heating rate; (**c**) compression amount × cooling rate; (**d**) heating rate × heating sealing temperature; (**e**) cooling rate × heating sealing temperature; (**f**) heating rate × cooling rate. Color variation (blue to red) indicates low to high bending strength.

To visually represent the influence of multi-factor coupling on the tensile strength, three-dimensional response surfaces and contour plots were drawn based on regression models for different combinations of process parameters, as shown in Figure 5. The factors not examined remained at the central level to highlight the influence patterns of the pairwise interaction terms on the tensile strength. Overall, within the experimental range, the tensile strength showed a single-peak trend that first increased and then decreased as each factor changed, and the response surface presented a significantly curved saddle-shaped or dome-shaped form, which was consistent with the statistically significant results of the quadratic term.

(1) Interaction between compression amount (A) and heating sealing temperature (B) (AB) 

The interaction between the compression amount (A) and heating sealing temperature (B) (AB) is shown in Figure 5a,b. When the cooling rate and heating rate are at the central level, the slope of the response surface on the A-B plane is large, indicating that the interaction between the compression amount and heating sealing temperature is the most sensitive to the tensile strength. At low compression amounts (A is smaller), as the heating sealing temperature increases from 80 °C to the central level, the tensile strength increases rapidly; when the temperature further increases, due to the low resin viscosity and excessively fast curing rate, the resin loss and thermal residual stress increase, and the strength decreases instead. Conversely, at higher compression amounts, moderately increasing the heating sealing temperature is beneficial for reducing the resin viscosity, promoting wetting and venting, but when both are too high, it causes severe resin-pooling and fiber-buckling defects, resulting in a significant decrease in strength. Therefore, there is a “synergistic optimal” area between A and B: a compression amount slightly above the central value and a heating sealing temperature slightly above the central temperature can achieve the maximum tensile strength.

(2) Interaction between compression amount (A) and cooling rate (D) (AD)

The interaction between the compression amount (A) and cooling rate (D) (AD) is shown in Figure 5c,d. On the A–D plane, the response surface also presents a clearly curved morphology. For smaller compression amounts, appropriately reducing the cooling rate helps to reduce the thermal residual stress at the fiber/resin interface after curing, avoiding microcracks and interface delamination caused by rapid cooling, and the tensile strength increases with the decrease in the cooling rate; but when the compression amount is small, excessively slow cooling does not significantly improve the fiber volume fraction and porosity, resulting in limited strength improvement. For larger compression amounts, high cooling rates are superimposed with high fiber volume fractions, significantly amplifying the residual stress caused by the mismatch of thermal expansion coefficients, resulting in a sharp decrease in strength. Therefore, there is also a clear synergistic control area between the compression amount and cooling rate: at medium compression amounts (approximately 0.07–0.10 mm) and moderately low cooling rates (approximately 4–6 °C/min), it is possible to effectively release residual stress while ensuring the degree of densification, thereby obtaining a higher tensile strength.

(3) Interaction between heating sealing temperature (B) and cooling rate (D) (BD)

The interaction between the heating sealing temperature (B) and cooling rate (D) (BD) is shown in Figure 5e,f. The slope of the B–D interaction surface is slightly lower than those of AB and AD but still shows a significant influence. Overall, at lower heating sealing temperatures, the influence of changing the cooling rate on the tensile strength is relatively mild; while at higher heating sealing temperatures, the influence of the cooling rate is amplified. The combination of high temperature and rapid cooling will cause greater thermal residual stress and curing shrinkage mismatch, resulting in a significant decrease in strength. In other words, the higher the heating sealing temperature, the stricter the control requirements for the cooling rate, and a relatively slow and uniform cooling regime is needed to avoid interface microcracks and interlamellar delamination.

(4) Comprehensive optimization and optimal process window

By considering the main effects, interaction effects, and response surface morphology of each factor, it can be seen that there are reasonable process windows for A, B, C, and D. Within the variable range of the Box–Behnken design, the optimal process parameters predicted by the model are: compression amount: 0.067 mm; heating sealing temperature: 114.927 °C; heating rate: 1.353 °C/min; cooling rate: 4.578 °C/min. At this time, the theoretical maximum value of the tensile strength is 2286.52 MPa, with a 95% confidence interval (CI) ranging from 2245.10 MPa to 2327.94 MPa. The validation experiment yielded an average value of 2291.26 MPa, which falls well within this 95% CI, indicating the high reliability and predictive accuracy of the regression model. Considering the operability of actual engineering control, the parameters are set as a compression amount of 0.07 mm, a heating sealing temperature of 114.93 °C, a heating rate of 1.35 °C/min, and a cooling rate of 4.58 °C/min as the optimal process parameters to prepare CFRP unidirectional plates and conduct three sets of repeated verification tests. The measured average tensile strength is 2291.26 MPa, which is highly consistent with the predicted value, with a deviation within the engineering allowable range. This further proves that the established response surface model has good predictive ability and practical value.

**Figure 6:**
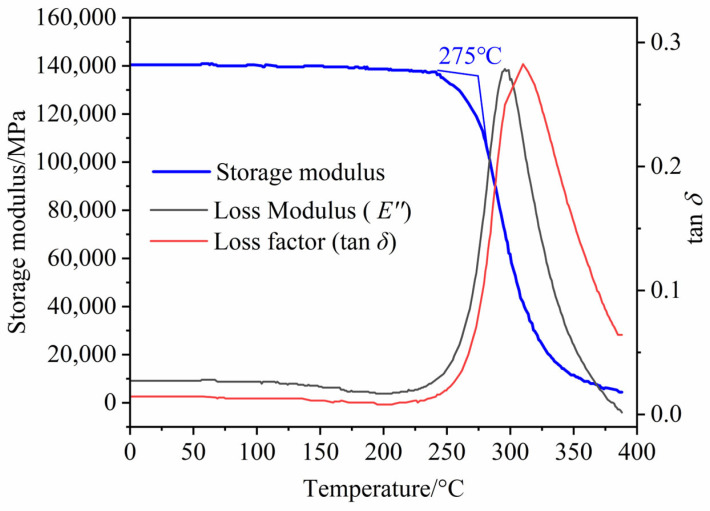
DMA curve of carbon fiber/dual-matrix resin CFRP unidirectional plate under optimal process.

Moreover, the dynamic thermal mechanical analyzer (DMA, DMAQ850, New Castle, DE, USA) was used to verify the dynamic thermal mechanical properties of the carbon fiber double-matrix resin CFRP unidirectional plates under this process. Figure 6 shows the dynamic mechanical behavior of the carbon fiber double-matrix resin composite material with temperature changes, which is mainly characterized by the storage modulus (E’), loss modulus (E″), and loss factor (tan β). Among them, the storage modulus reflects the stiffness of the material in the elastic deformation stage, while the loss factor reflects the ability of the material to dissipate energy and the damping characteristics. From the figure, it can be seen that in the low-temperature and medium-temperature regions (0 °C~250 °C), the storage modulus of the composite material remains at a high level of approximately 140 GPa, and the curve shows excellent plateau characteristics. This indicates that the high crosslink density network formed by the double-matrix resin limits the movement of molecular chains, giving the material excellent thermal dimensional stability and rigidity. As the temperature further increases, the storage modulus begins to show a turning point and drops sharply at 275 °C. This temperature point corresponds to the starting point of the resin matrix’s transition from the glass state to the highly elastic state, meaning that the polymer chain segments begin to freeze and thaw, and the material rigidity significantly decreases. Therefore, 275 °C can be regarded as the safe service upper-limit temperature for this composite material in load-bearing structure applications. The peak temperature of the loss factor (tan β) curve is usually defined as the glass transition temperature (Tg) of the material. The test results show that the Tg of this composite material is as high as 312 °C, demonstrating extremely excellent heat resistance. This high-Tg characteristic is attributed to the rigid molecular framework of the double-matrix resin and the effective restriction of the carbon fiber on the polymer chain segment movement. In conclusion, the CFRP unidirectional plates prepared under the optimal process parameters obtained using the response surface have excellent mechanical performance retention below 275 °C, suitable for aerospace structural components with strict requirements for high-temperature resistance and rigidity.

### 3.4. The Application of the Optimal Process in Aircraft Shell Manufacturing

**Figure 7:**
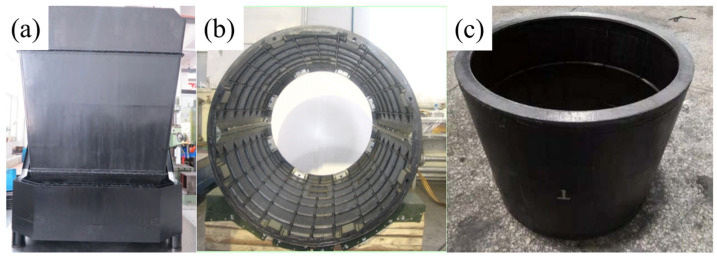
Engineering application of BMI–CFRP unidirectional laminates processed under optimal parameters: (**a**) square reinforced housing assembly; (**b**) the cylindrical reinforced shell structure of the aircraft; (**c**) cylindrical reinforced shell component.

To verify the engineering applicability and stability of the hot-melt pre-impregnation—fiber-winding—metal-mold-forming process for the BMI/CF composite material in high-temperature bearing structures, this study directly applied the optimal process parameters obtained by the response surface method (compression amount: 0.07 mm; hot-bonding temperature: 114.93 °C; heating rate: 1.35 °C/min; cooling rate: 4.58 °C/min) to the trial production of typical aircraft reinforcement shell structures. Figure 7 shows the actual application of the BMI-CFRP unidirectional plate prepared based on the optimal process as a reinforcement shell or fairing product in aircraft. During the trial production process, the resin flow/exhaust behavior during the mold-pressing and hot-bonding stages was consistent with the calibration results of the unidirectional plate process window, the part was easily demolded, the reinforcement strips were formed completely, and the surface continuity was good, demonstrating the controllability and repeatability of this parameter combination in the engineering forming process.

The surface porosity of the fabricated aircraft BMI-CFRP reinforcement shell was controlled below 0.8%, and the fiber volume fraction was increased to approximately 62%, which was significantly better than that of the traditional hot-press tank-forming process (about 55%). Through room-temperature and high-temperature tensile tests, the reinforcement shell product maintained a stable tensile strength around 2300 MPa, the thickness error of the part was controlled within ±0.05 mm, and the weight was reduced by approximately 38% compared to traditional aluminum alloy structures, with a heat deformation reduction of 41%. In addition, thanks to the high glass transition temperature and excellent thermal oxidation stability of the dual-matrix resin system, the composite shell maintained a mechanical performance retention rate of over 93% after 200 h of testing under high-temperature cycling (150–200 °C) and a humid heat environment (RH: 90%, 70 °C), demonstrating excellent environmental service stability and meeting the long-service requirements of high-speed aircraft. In summary, the multi-factor optimization process proposed in this paper not only improves the structural strength and dimensional accuracy of the composite material but also significantly improves the consistency and environmental adaptability of the part, providing a reliable engineering path and theoretical support for the lightweight structural design of composite materials in high-speed aircraft, reusable launchers, high-temperature unmanned aircraft, and other fields.

Despite the demonstrated effectiveness of the proposed manufacturing route, certain limitations should be acknowledged. Firstly, regarding the geometric applicability, the matched-die-molding process relies on rigid metal molds with high clamping pressures. While highly efficient for cylindrical or conical stiffened shells, this method faces challenges when fabricating structures with complex undercuts or variable-thickness topological optimizations, where demolding becomes difficult compared to flexible vacuum-bagging techniques. Secondly, concerning the service conditions, this study primarily focused on static mechanical properties and isothermal aging. However, hypersonic vehicles operate under cyclic thermomechanical loading environments. The fatigue damage evolution and interface degradation mechanisms of the BMI-CFRP under such dynamic coupling conditions were not covered in this work and will be the focus of our future investigations.

## 4. Conclusions

This study was aimed at the forming requirements of high-temperature lightweight bearing structures for hypersonic aircraft, and we proposed and verified an integrated manufacturing process route of “hot-melt prepregging—fiber winding—metal mold forming” for BMI-CFRP. Using a unidirectional plate as the process window calibration carrier, pre-experiments and single-factor tests were adopted to define the feasible parameter range, and the Box–Behnken response surface method was combined to achieve the global optimization of the key forming parameters, establishing a reliable process prediction model.

The results show that the compressive amount (A), heat-bonding temperature (B), heating rate (C), and cooling rate (D) all exhibit a single-peak response to tensile strength, with a significance ranking of B > D > A > C. Among them, the interaction between A and B and between A and D is extremely significant, and the interaction between B and D is significant. This indicates that there is a clear coupling between compaction—resin flow/infiltration—residual stress release. The established quadratic regression model is significant and has a good fit (*p* < 0.001; lack of fit is not significant, *p* = 0.5021; R^2^ = 0.974; R^2^Adj = 0.949), which can be used for parameter prediction and quantification of the process window. The optimal combination predicted by the model is A = 0.067 mm, B = 114.93 °C, C = 1.35 °C·min^−1^, and D = 4.58 °C·min^−1^, and the verification test results are highly consistent with the prediction. Microscopic characterization shows that under the optimal conditions, the interlayer bonding is denser, and the pores are significantly reduced, supporting the defect control mechanism for strength improvement; the DMA results further indicate that the material has thermal–mechanical-stability-matching high-temperature applications. Transferring the optimal parameters to the reinforcement shell sample preparation can achieve stable forming with a fiber volume fraction > 60% and a porosity < 1%, verifying the transferability and engineering applicability of this process and parameter window to complex structural components.

In summary, this study not only determined the independent optimal ranges of each process parameter but, more importantly, also revealed the interaction effects between the parameters and obtained the global optimal process combination. The established prediction model provides scientific theoretical guidance and process control basis for the hot-melt prepreg forming of BMI/CF composites, can effectively improve the forming accuracy and mechanical properties of the products, and has important engineering application value.

## Figures and Tables

**Table 1:** Process parameters of prepreg.

Procedure	Value
Coating temperature (°C)	110
Coating speed (m/min)	6
Coating pressure (N)	20
Infiltration temperature (°C)	120
Infiltration speed (m/min)	3
Infiltration pressure (N)	30

**Table 2:** Single-factor experiment.

Serial Number	Test Conditions	Observations
1	Heating sealing temperature: 120 °C Heating rate: 1.5 °C/min Cooling rate: 10 °C/min	Amount of compression (mm): 0.05; 0.1; 0.15; 0.2; 0.25
2	Amount of compression: 0.15 mm Heating rate: 1.5 °C/min Cooling rate: 10 °C/min	Heating sealing temperature (°C): 80; 100; 120; 140; 160
3	Amount of compression: 0.15 mm Heating sealing temperature: 120 °C Cooling rate: 10 °C/min	Heating rate 0.5 (°C/min): 1; 1.5; 2; 2.5
4	Amount of compression: 0.15 mm Heating sealing temperature: 120 °C Heating rate: 1.5 °C/min	Cooling rate (°C/min): 2; 6; 10; 14; 18

**Table 3:** RSM Test Factor Levels.

Levels	Factors			
	A Amount of Compression (mm)	Heating Sealing Temperature (°C)	Heating Rate 0.5 (°C/min)	Cooling Rate (°C/min)
−1	0.05	80	0.5	2
0	0.10	100	1.0	6
1	0.15	120	1.5	10

**Table 4:** Experimental results of response surface methodology.

No.	Amount of Compression (mm)	Heating Sealing Temperature (°C)	Heating Rate (°C/min)	Cooling Rate (°C/min)	Tensile Strength (Mpa)
1	0.05	80	1	6	507.99
2	0.15	80	1	6	1198.62
3	0.05	120	1	6	2247.24
4	0.15	120	1	6	2095.06
5	0.1	100	0.5	2	1766.22
6	0.1	100	1.5	2	1887.23
7	0.1	100	0.5	10	1322.69
8	0.1	100	1.5	10	1136.78
9	0.05	100	1	2	1906.63
10	0.15	100	1	2	1600.11
11	0.05	100	1	10	796.3
12	0.15	100	1	10	1356.35
13	0.1	80	0.5	6	600.23
14	0.1	120	0.5	6	2001.87
15	0.1	80	1.5	6	808.23
16	0.1	120	1.5	6	1916.75
17	0.05	100	0.5	6	1587.83
18	0.15	100	0.5	6	2000.54
19	0.05	100	1.5	6	1597.25
20	0.15	100	1.5	6	1765.2
21	0.1	80	1	2	606.77
22	0.1	120	1	2	2174.89
23	0.1	80	1	10	507.52
24	0.1	120	1	10	1502.44
25	0.1	100	1	6	1877.57
26	0.1	100	1	6	2026.68
27	0.1	100	1	6	2117.62
28	0.1	100	1	6	2209.55
29	0.1	100	1	6	2076.15

**Table 5:** Regression analysis table.

Source	Sum of Squares	Degrees of Freedom	Mean Square	F-Value	*p*-Value
Model	8,662,866.94	14	618,776.21	38.10	0.0000
Amount of compression	157,011.71	1	157,011.71	9.67	0.0077
Heating sealing temperature	4,952,248.75	1	4,952,248.75	304.96	0.0000
Heating rate	2350.32	W1	2350.32	0.14	0.7093
Cooling rate	918,406.07	1	918,406.07	56.56	0.0000
AB	177,582.17	1	177,582.17	10.94	0.0052
AC	14,976.86	1	14,976.86	0.92	0.3532
AD	187,735.89	1	187,735.89	11.56	0.0043
BC	21,479.83	1	21,479.83	1.32	0.2694
BD	82,139.56	1	82,139.56	5.06	0.0411
CD	23,549.97	1	23,549.97	1.45	0.2485
A^2^	150,155.44	1	150,155.44	9.25	0.0088
B^2^	1,394,027.16	1	1,394,027.16	85.85	0.0000
C^2^	223,647.85	1	223,647.85	13.77	0.0023
D^2^	1,112,008.04	1	1,112,008.04	68.48	0.0000
Residual error	227,343.56	14	16,238.83		
Missing item	167,018.00	10	16,701.80	1.11	0.5021
Error	60,325.56	4	15,081.39		
Total	8,890,210.49	28			

## Data Availability

The original contributions presented in this study are included in the article. Further inquiries can be directed to the corresponding author.

## Abbreviations

The following abbreviations are used in this manuscript:

BBD	Box–Behnken design
BDM	4,4′-Bismaleimidodiphenylmethane
BMI	Bismaleimide
CCD	Central composite design
CF	Carbon fiber
CFRP	Carbon fiber-reinforced polymer
CNC	Computer numerical control
CTE	Coefficient of thermal expansion
DABPA	2,2′-Diallylbisphenol A
DMA	Dynamic mechanical analysis
DSC	Differential scanning calorimetry
FRP	Fiber-reinforced polymer
RH	Relative humidity
RSM	Response surface methodology
SEM	Scanning electron microscopy
Tg	Glass transition temperature
